# Is head–shaft angle a valuable continuous risk factor for hip migration in cerebral palsy?

**DOI:** 10.1007/s11832-016-0774-0

**Published:** 2016-10-12

**Authors:** Sanjay Chougule, John Dabis, Aviva Petrie, Karen Daly, Yael Gelfer

**Affiliations:** 1 East Surrey Hospital NHS Trust, Redhill, RH1 5RH UK; 2 St Georges Hospital NHS Foundation Trust, London, SW17 OQT UK; 3 University College London NHS Foundation Trust, London, WC1X 8LD UK; 4 21 Arcadian place, London, SW18 5JF UK

**Keywords:** Cerebral palsy, Hip migration, Head shaft angle

## Abstract

**Background:**

Reimer’s migration percentage (MP) is the most established radiographic risk factor for hip migration in cerebral palsy (CP), and it assists surgical decision-making. The head–shaft angle (HSA) measures the valgus of the head and neck in relation to the shaft and may also be a useful predictor of hip migration at a young age. This study first defined normal values and investigated whether the head–shaft angle (HSA) is a continuous risk factor for hip migration in CP.

**Methods:**

Three hundred and fifty AP pelvic radiographs of 100 consecutive children comprising the hip surveillance programme in our region were analysed for MP and HSA. Inclusion criteria were children with spastic CP and Gross Motor Function Classification System (GMFCS) levels of III–V, along with a minimum follow-up of 5 years. The mean age was 8.8 (range 3–18) years and the mean follow-up time was 7.5 (range 5–10) years. Radiographs of 103 typically developing children (TDC) were selected for the control group. The reliability of the measurements was determined. A random effects analysis was used to assess the relationship between MP and HSA for all data and for MP > 40 %.

**Results:**

The TDC cohort had a mean HSA of 157.7° whilst that for the CP cohort was 161.7°. The value declined with age in both groups but remained consistently higher in the CP group. A random effects analysis considering the longitudinal data showed that there was no significant effect of HSA on MP. Similarly, when excluding CP patients with MP < 40 %, there was no significant effect of HSA on MP.

**Conclusions:**

This study found no correlation between HSA and hip migration in children with CP in this age group. Using the HSA as a routine radiographic measure in the management pathway across childhood does not offer any added value. Early enrolment onto the hip surveillance programme could offer a better prediction of hip migration using the HSA at a very young age.

**Level of evidence:**

II retrospective prognostic study.

## Introduction

Hip displacement is one of the two most common musculoskeletal manifestations in cerebral palsy (CP) [1]. The incidence of progressive lateral displacement of the hip is ≤35 % across all children with CP [2]. Contracture of the muscle–tendon units, bony torsional deformities and joint instability probably all contribute to this progressive deformity [3]. The risk of hip migration increases with increasing Gross Motor Function Classification System (GMFCS) level [2, 4].

Early detection of hip migration requires a combination of regular clinical examinations and radiographs. Radiographic hip surveillance programmes have improved the outcome for children with CP, as the introduction of a preventative programme at a younger age has led to a reduction in salvage surgery for painful dislocated hips [3, 5, 6]. The migration percentage (MP) as described by Reimers is the gold standard method for assessing radiographs [5, 7, 8, 9, 10]. The recommended practice is close follow-up for hips ‘at risk’ (MP 33–40 %) and surgical intervention, in the form of soft-tissue or bony procedures, for MP > 40 % [11].

The neck–shaft angle (NSA) increases in a stepwise fashion from GMFCS I to V [12]. It assesses coronal plane deformity, but there is a substantial measurement error when the effect of the femur anteversion is not considered [13]. The HSA describes an angle that is not only attributed to the neck and shaft but also to the relationship between the neck and femoral head. This relationship is increased in children with cerebral palsy and in hips requiring surgical correction [13]. The prognostic value of the HSA in children with CP has recently been investigated and it was suggested to be a predictor for hip displacement [14, 15].

Our first aim was to define the normal distribution of HSA with age in a cohort of typically developing children (TDC). This was recently determined for children under 7 years old [16].

The secondary aim of this study was to compare the HSA in children with CP to the normal population and then to ascertain whether the HSA is of value for continuously predicting lateral migration of the hip. For this, we subdivided our CP cohort into those with and without migration (defined as MP > 40 %).

## Methods

Review of the PACS records identified a consecutive cohort of 103 normal AP pelvic radiographs that had been taken in A&E during a 6-month period. The age range was 0–13 years.

The study group consisted of a consecutive group of 100 children with CP who were enrolled into the hip surveillance programme in an area serving both a district general hospital and a tertiary referral centre in South West London and Surrey, with a population of 1.5 million. The developmental paediatrician made the diagnosis of CP and GMFCS level. The hip surveillance programme includes an annual AP radiograph of the pelvis and biannual radiographs for hips, which are noted to be migrating or ‘at risk’. The first radiograph is done as soon as the diagnosis is made, although, due to the population diversity and continuous turnover in the London area, the age at presentation and the compliance with the programme can vary. The supine positioning of the patient for the radiograph, as per hospital protocol, has been standardised and previously audited to avoid variability in positioning and anteversion.

Inclusion criteria included all children with spastic CP and GMFCS III–V who were enrolled in the hip surveillance programme and had a minimum follow-up of 5 years. Exclusion criteria included closed growth plates, previous hip surgery, nonspastic CP and hemiplegia. The data from one hip was randomly excluded from the analysis.

**Fig. 1:**
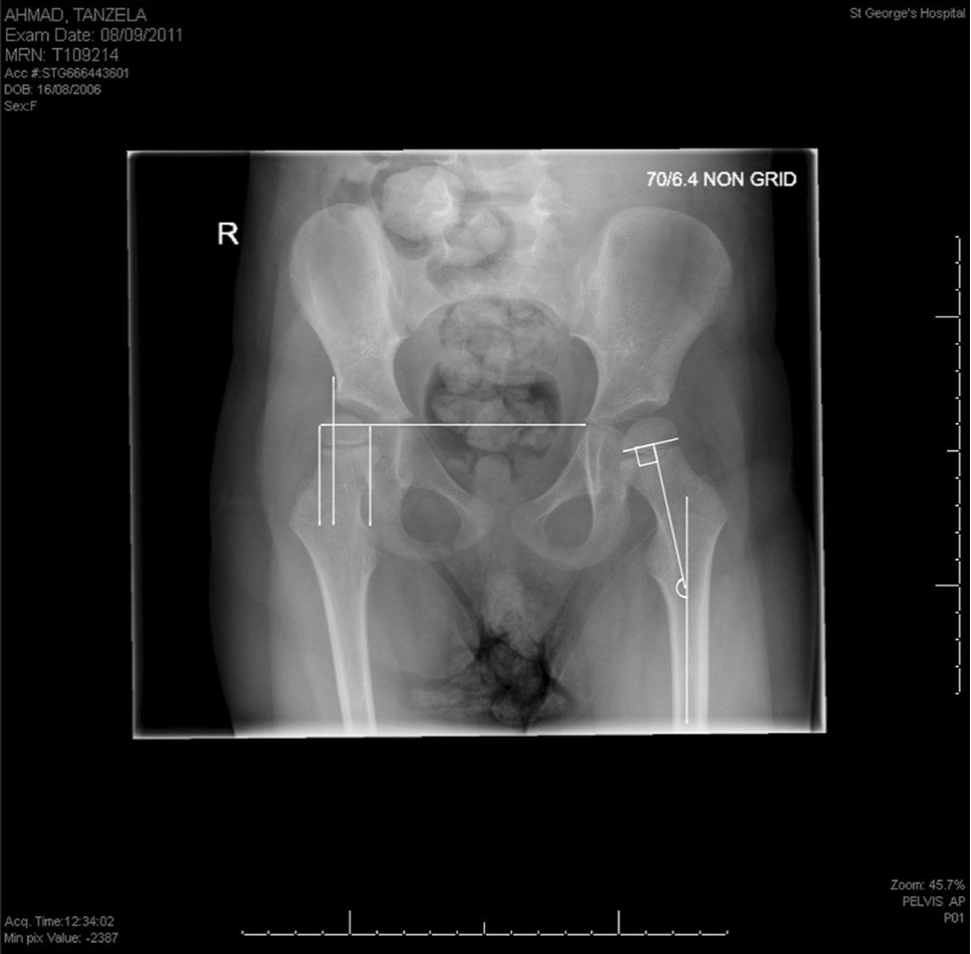
Measurement of the MP on the right hip and HSA on the left hip

The hip migration was measured using the Reimers MP method [7] and the HSA was measured according to Southwick [17] (Fig. 1). These are acceptable measures that have been described previously [7, 18–22]. The radiographs from the study group were examined to assess the relationship between HSA and MP. The analysis was done for the entire group and for radiographs with MP > 40 %.

A reliability study was conducted to assess intraobserver variability of HSA and MP in a single observer (author SC). Fifty randomly selected hip radiographs from the CP database were used by a blinded examiner to measure the HSA and MP. The measurements were conducted twice, a week apart. One side was randomly excluded from the analysis following completion of the data collection. In order to assess repeatability, the Bland and Altman approach was used. The British Standards Institution repeatability coefficient and Lin’s concordance correlation coefficient were calculated, and a paired *t* test was performed to investigate a systematic effect.

A random effects analysis, incorporating age as a covariate, was used for the longitudinal data to assess the relationship between MP (the dependent variable) and HSA. This analysis was performed on all the data and repeated for MP > 40 %. Linear regression analysis was used to assess the relationship between HSA and age, separately for the normal and the CP patients, using baseline measurements for one side (chosen randomly), for both groups, and baseline measurements for the CP patients. This was followed by multivariable regression analysis with HSA as the dependent variable and age and group as the explanatory variables, with the interaction between age and group taken as a covariate.

Statistical analyses were performed using Stata (Stata Corp. 2013) and SPSS (IBM Corp. 2013). The significance level was set at 5 %.

## Results

The reliability study showed both HSA and MP measurements to be reliable. For HSA, Lin’s concordance correlation coefficient was estimated as 0.88 [95 % confidence interval (CI) 0.79–0.93] and the British Standards Institution correlation coefficient, estimating the maximum likely difference between repeat measurements, was 7.6°, with no evidence of a systematic difference between the repeat measurements (*p* = 0.83). For MP, Lin’s concordance correlation coefficient was estimated as 0.96 [95 % confidence interval (CI) 0.92–0.98] and the British Standards Institution correlation coefficient was 6.5 %, with no evidence of a systematic difference between repeat measurements (*p* = 0.12).

The control group of 103 TDC, mean age 5.9 years (SD 3.2 range 0–13 years), had a mean HSA of 157.7° (SD 7.1°, range 139–175°).

The CP group comprised 100 patients, mean age at first radiograph 8.8 (SD 4.3, range 3–18) years, mean follow-up time 7.5 (range 5–10) years, and mean number of radiographs 3.3 (range 2–8). Seventeen patients had MP > 40 % at baseline, and mean HSA was 161.7° (SD 10.1°, range 131–184°).

**Fig. 2:**
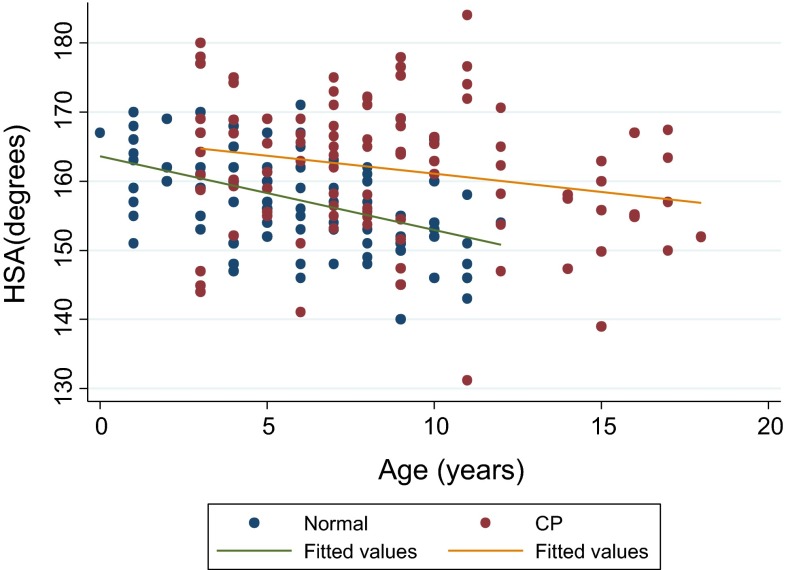
Scatter diagram for HSA versus age in the cohort of CP patients versus the TDC population, including regression lines

Three hundred and fifty radiographs were included in the study group. Multivariable regression analysis of the baseline data showed that there was no evidence of an interaction between age and group (*p* = 0.10), that HSA significantly declined with age in both groups (regression coefficient 0.71°, 95 % CI −1.02° to −0.40°, *p* < 0.001) and that the mean value of the HSA was significantly higher in the CP population than in the normal population, with a mean difference of 6.4° (95 % CI 4.0–8.8°, *p* < 0.001), Fig. 2.

**Fig. 3:**
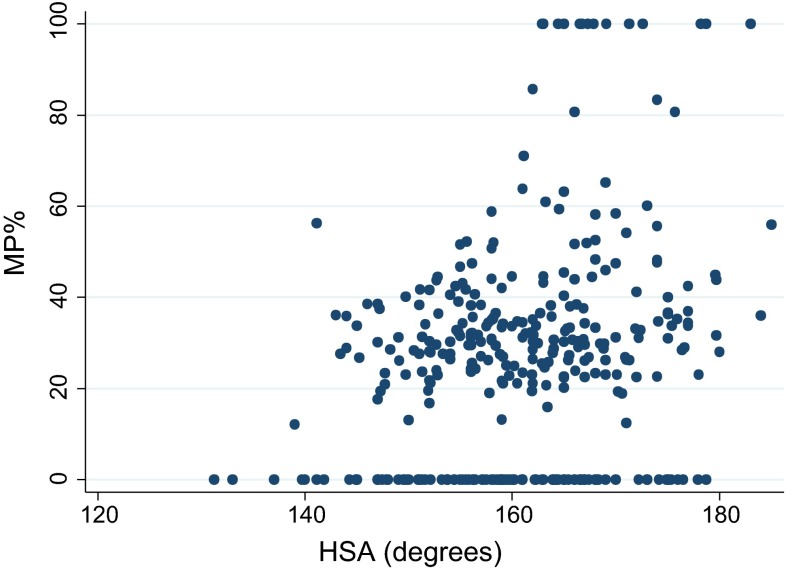
Scatter diagram for HSA versus MP in the cohort of CP patients

The random effects analysis considering the longitudinal data showed that there was no significant effect of HSA on MP (estimated regression coefficient 0.41 % (95 % CI −0.08 to 0.91 %, *p* = 0.10) after adjusting for age (which was not significant, *p* = 0.24) (Fig. 3).

**Fig. 4:**
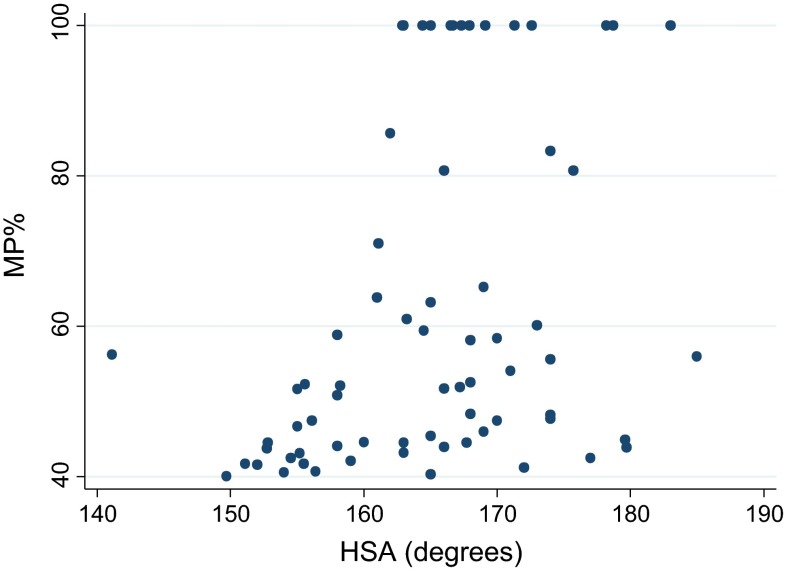
Scatter diagram for HSA versus MP in the cohort of CP patients with MP >40 %

Similarly, when excluding CP patients with MP < 40 %, there was no significant effect of HSA on MP (estimated regression coefficient 0.07 % (95 % CI −0.15 to 0.28 %, *p* = 0.55) after adjusting for age (which was not significant, *p* = 0.77) (Fig. 4).

## Discussion

At birth the normal femur is anteverted. There is a failure of this anteversion to resolve with growth in children with CP: progressive deformity may be due to a combination of abnormal biomechanics and neuromuscular imbalance. Understanding the cause of the proximal femur deformity in cerebral palsy and its significance can guide management and care. Many investigators have studied risk factors for hip displacement, and there is much controversy in the literature regarding the effect of proximal femoral anatomy on hip migration.

Robin et al. [4] studied the relationship between proximal femoral anatomy, GMFCS and hip migration. Clinical femoral neck anteversion and radiographic NSA were found to be increased in children with cerebral palsy compared to TDC. The increase in anteversion occurred in a stepwise manner with increasing GMFCS level, with a pattern similar to the changing MP. The authors concluded that the abnormal shape of the proximal femur could explain hip displacement in CP. The HSA was not included in the analysis.

Measurement of the NSA is largely influenced by femoral anteversion [21], and the hip needs to be positioned in maximum internal rotation in order to compensate for femoral anteversion [4, 23]. The protocol in our institution, as in most protocols, adopts a standard position with the child’s limbs in neutral hip rotation and the patellae facing upwards [3, 5, 24]. The measured NSA has a predictable error, which potentially prevents its use as another predictor of hip migration. The HSA incorporates the position of the head compared to the neck and is less influenced by femoral anteversion [13, 15, 25].

Several studies have published data on the distribution of NSA in normally developed children [23, 26–29]. Van der List et al. assessed the HSA in TDC at ages 2, 4 and 7 years and compared the decline to HSA in CP children. This study defines the relationship of age to HSA in TDC under 14 years.

Lee et al. [12] studied the clinical relevance of proximal femoral deformity in patients with CP. 384 patients with CP were studied for GMFCS level and radiographic measurements of NSA, HSA and MP. The NSA showed higher correlation than HSA with MP, which led the authors to conclude that the NSA better reflects the likelihood of hip migration than the HSA. Our study cohort did not show any effect of HSA on hip migration.

Van der List et al. [14] documented GMFCS levels and measured the radiographs of 50 children for HSA and MP, 35 of whom had GMFCS III, IV and V at three age intervals (age 2, 4 and 7), to determine the predictors for hip displacement at age 7. They found GMFCS to be an important predictor at age 2 and, at this age, HSA played a small additional role. The HSA was not found to be a predictor at age 4 and the outcome was completed at age 7. The study included both hips in the analysis and was lacking normal reference values. The repeatability of the examiner was not examined.

Hermanson et al. [15] assessed the HSA as a risk factor for hip displacement in 145 children with CP. The risk ratio for developing hip displacement independently of age and GMFCS was determined. HSA was found to be a risk factor for developing hip displacement of >40 % at 5 years. Normal reference values were not offered and information regarding repeatability and number of examiners was not available. A lower repeatability than reported in the literature would affect the risk ratio determined.

The mean HSA in our study was 161.7°, which was lower than 166° reported by Hermanson et al. [15]. The mean age at first radiograph was also higher in our study (8.8, range 3–18) compared to Hermanson et al. (3.5, range 0.6–9.7). As the study population in the London area is fluctuating and the model hip surveillance programme starting at age 2 is more challenging to achieve, the mean age at first radiograph was higher. The significance of the HSA in predicting migration might be restricted to the younger age group and is nonprogressive. Our study showed the HSA to decrease with age for both the TDC and the CP group; thus, the lower mean HSA value in the Hermanson paper compared to ours might be age-related.

We found that the HSA was higher in the CP group than in the TDC, which supports the results reported in previous publications [13]. This study found no significant effect of HSA on MP either for the CP group as a whole or for the subgroup with MP > 40 % for this age group.

It is important to determine the proximal femoral pathology to consider appropriate surgical correction. Varus osteotomy decreases both the NSA and the HSA and, as the immediate postoperative varus can reduce with time [30–32], the cause and direction of this abnormal growth needs to be more fully understood. We have not found grounds to recommend HSA measurements complementing MP to aid in decision-making at any age. Early enrolment onto the hip surveillance programme and continuous monitoring might be able to offer better prediction of hip migration.

### The study limitations

The study population is a combination of a district general hospital (DGH), representing a community population, and a tertiary referral centre population. The hip surveillance programme follows the same protocol, but as the population in this urban area fluctuates, extrapolation and generalisation should be done with caution.

The study group included patients with GMFCS III, IV and V combined, hence information regarding the behaviour of each group separately was not available. Lee et al. [12] found no significant difference in the HSA between GMFCS III, IV and V, and there is no reason to believe that the HSA in one subgroup will show a different distribution compared to the distributions in the other subgroups.

No further clinical information regarding the patients was incorporated, such as the level of spasticity, comorbidities, and clinical examination findings such as hip range of motion.

### The study strengths

This was a longitudinal-based study with a standardised protocol for patient positioning and radiographic measurements. A high reliability was calculated for the measurements and it presented normal reference values of HSA for TDC, which were compared with those for the CP cohort. Data independence was assured by randomly choosing one hip for analysis.

This study found no correlation between HSA and hip migration in children with CP at this age group, and therefore no indication for HSA to be used as a useful adjunct in the hip surveillance programme in CP throughout childhood. The use of this tool in the very young age group could offer more value.
